# Central bank communication, shadow banking, and bank risk-taking: Theoretical model and PVAR empirical evidence

**DOI:** 10.1371/journal.pone.0275110

**Published:** 2022-09-28

**Authors:** Jing Zhang, Shuai Chen, Honglei Liu

**Affiliations:** School of Finance, Shandong University of Finance and Economics, Jinan, Shandong, P.R. China; BeiHang University School of Economics and Management, CHINA

## Abstract

The rapid development of Chinese shadow banking, which relies on the banking system, significantly impacts nonneutral bank risk-taking. Central bank communication also plays a vital role in risk prevention and mitigation in the banking system in a modern central banking system. To address the issues of central bank communication, shadow banking, and bank risk-taking, we construct a new theoretical model that includes shadow banking based on the M-S model. Based on the central bank written communication index measured by the Monetary Policy Implementation Report 2009–2019 and balanced panel data of 35 listed banks, we design an empirical PVAR model. This study finds that central bank communication regarding domestic easing economy and policy positively affects bank risk-taking, while central bank communication and mechanism exists in China. Moreover, the growth rate of shadow banking size also positively encourages bank risk-taking. Furthermore, central bank communication regarding foreign policy is negatively related to bank risk-taking in the robustness test, supporting the above findings. The analysis of equity heterogeneity shows that the positive effect of central bank communication on bank risk-taking in shadow banking is more pronounced in small and medium-sized shareholdings. Further analyzing the economic consequences of central bank communication and shadow banking on bank risk-taking, we find that banks’ risk-taking positively affects their share of noninterest income and total asset size.

## 1. Introduction

Nonneutral bank risk-taking has been of great importance to countries since it surfaced. The experience of the global crisis shows that the impact of financial innovation, typically represented by shadow banking, on banks’ risk-taking cannot be underestimated. The same period proposed a monetary policy bank risk-taking channel and focused on the continued easing of monetary policy and bank risk-taking. However, national monetary policy in recent years has shown a new trend of increasingly frequent central bank communication operations and increasingly prominent roles.

Establishing a modern central banking system worldwide is inseparable from a perfect central bank communication system. Automating and rationalizing monetary policy communication guides market participants’ expectations and facilitates improving the transmission mechanism of monetary policy. The People’s Bank of China has been taking the initiative to strengthen communication since 2020, gradually forming institutionalized communication methods such as announcing operational arrangements for medium-term lending facilities in advance and issuing press releases on policies. However, the long-established, institutionalized, authoritative, widely influential, and operationally independent communication method remains the quarterly mid-monthly release of the Report on the Implementation of China’s Monetary Policy (hereafter referred to as the Report). The Report received much attention. Many studies have clarified its essential impact on financial markets, business investment, and inflation expectations, affirming its positive role in the transmission mechanism of monetary policy [1, 2]. Central bank communication also play an essential role in preventing and defusing major systemic risks by influencing banks’ risk-taking. Unfortunately, this has not received enough attention from the academic community, as evidenced by the lack of research on central bank communication and bank risk-taking in China. However, the People’s Bank of China has continued to combat some of the irregular operational behaviors on the deposit side of banks since the fourth quarter of 2020 at the policy practice level. They use various information and communication methods to guide the entity’s interest rate expectations and influence asset-side decisions. Central bank communication naturally has a direct impact on the risk-taking appetite of banks. Therefore, the development of the current situation needs to be closely followed by academic research. It is necessary to carry out theoretical and empirical investigations on the central bank’s communication behavior concerning banks’ risk-taking.

However, shadow banking is closely related to risk-taking in banks, especially in China, where the relationship is even closer. Scholarly studies note that the new regulatory restrictions introduced by the regulatory authorities in China have facilitated the further development of shadow banking in banks in the context of the rapid growth of fintech [3]. Shadow businesses helps banks do away with their restrictions. They obtain more sources of funds through off-balance-sheet wealth management and deposit-like products and diversify the use of funds through asset securitization and channel-type businesses. These shadow banking behaviors are profoundly linked to banks’ risk-taking. The off-balance sheet operations in the U.S. banking industry have continued to increase the proportion of nondeposit liabilities and noninterest income [4].

This paper focuses on the theoretical and empirical investigation of the threefold relationship between central bank communication, shadow banking, and bank risk-taking, with the following main contributions. First, this study builds on the literature on the impact of central bank communication on bank risk-taking using the M-S theoretical model by incorporating shadow banking factors, and deriving an analysis of the important role of both central bank communication and shadow banking on bank risk-taking. We slightly expand the research on the effects of central bank communication as a policy tool, as well as the mechanism of central bank communication and exchange in the bank risk-taking channel of monetary policy, trying to touch the currently undiscovered marginal gap in theoretical research between central bank communication, shadow banking, and bank risk-taking. Second, the impact of central bank communication and shadow banking on bank risk-taking is not transient. The dynamic relationship between the three may be of more interest, so this paper uses the PVAR empirical method to test the long-term dynamic relationship. Third, banks with different ownership systems may not be as sensitive to central bank communication and may not be affected by shadow banking to the same extent, so this paper considers the effect of ownership heterogeneity. Fourth, what long-term economic consequences might be expected of central bank communication and shadow banking-influenced bank risk-taking? This paper explores the impact of bank risk-taking in the above endogenous system on bank profitability and asset size. Finally, this paper constructs a written communication index of the Chinese central bank on the domestic economy and policies and a written communication index of the Chinese central bank on foreign economies and policies, which to some extent provides a basis for the Chinese policy authorities to make decisions.

The rest of the paper is organized as follows. Section 2 presents the literature review. Section 3 constructs a novel economic model. Section 4 provides a brief description of our data sources. Section 5 reports our main empirical results, and Section 6 provides further analysis. Section 7 concludes.

## 2. Literature review

### 2.1. Unconventional monetary policy and bank risk-taking

Existing studies have confirmed the existence of a precrisis monetary policy bank risk-taking channel at the theoretical level [5]. At the empirical level [6, 7], this means that low-interest rates from accommodative monetary policy cause a rise in banks’ risk-taking. This type of research focuses on the impact of traditional monetary policy instruments on banks’ risk-taking. However, low-interest rates in many developed economies have been maintained for almost a decade after the financial crisis. They are expected to continue, with particular attention to the fact that central bank monetary policy has different effects during periods of financial stress compared with normal periods [8]. Some scholars have focused on the importance of unconventional monetary policy instruments by central banks in the zero interest rate floor dilemma and bank risk-taking. Brana, Campmas, and Lapteacru [8] use a dynamic panel model of threshold effects to study the impact of monetary policy, particularly unconventional monetary policy, on bank risk-taking behavior in Europe over the period 2000 to 2015. They find that low- interest rates and increased central bank liquidity through accommodative monetary policy have a nonlinear deleterious effect on bank risk. Matthys, Meuleman and Vennetb [9] used bank-firm level corporate syndicated loan data to construct a VAR model to identify bank risk-taking due to unconventional monetary policies in the U.S. from 2008 to 2015. An accommodative monetary environment was associated with overall lower loan spreads.

### 2.2. Central bank communication and bank risk-taking

The predominance of central bank communication in the unconventional monetary policies of national central banks has attracted the attention of scholars. They have launched a deep inquiry into the effectiveness of central bank communication and a communication approach based on clarity and transparency [10–12], tone of voice mood [13–15], language style [16], and verbal and written tools [17, 18]. Among them, written forms of communication such as the Monetary Policy Implementation Report, the Financial Stability Report, and the Financial Statistics Report, and press releases have received much attention and research, mainly around how to guide interest rate expectations [17, 19–21], exchange rate expectations [22], and inflation expectations [23]. In particular, a large number of studies have addressed the effects of financial market stability [2], financial asset prices [1, 19], and financial asset returns [20, 21, 24, 25]. In addition, a small number of scholars have started to study the impact of firms [26] or macroeconomic operations [18, 27]. Some scholars have also considered central bank communication’s monetary policy transmission perspective. However, existing studies mainly focus on traditional transmission channels [22, 28, 29] and cross-country spillover transmission channels. There is less empirical evidence involving bank risk-taking channels. However, since Borio and Zhu [5] proposed the bank risk-taking channel, they have identified central bank communication and the communication mechanism as one of the three main mechanisms of action, affecting banks’ risk-taking from a theoretical and practical point of view.

Blinder [30] was the first to show the transparency effect of central bank communication and exchange. In other words, it help banks more accurately predict the behavior of the monetary authority, reducing the risk premium while increasing bank risk-taking. Borio and Zhu [5] also proposed the "insurance effect" of central bank communication and exchange based on the above. The central bank’s reaction function can transmit the asymmetric impact of interest rate changes, which can effectively block the downside risk of the economy and reduce bank risk-taking. Later, based on the theory constructed by previous authors, Li and Gao [31] introduced the M-S model of Morris & Shin [32] (a theoretical model of public information affecting social welfare) to derive a proof of the effect of central bank disclosure on commercial banks’ expectations. They pointed out that central bank communication positively affects banks’ risk preferences.

### 2.3. Shadow banking and bank risk-taking

In summary, it is clear that central bank communication, which naturally exists with the creation of central banks, has received great attention in the last decade or so and is seen as an innovative and unconventional monetary policy tool [30]. A major innovation in the financial sector at the same time as central bank communication is shadow banking. Due to the financial crisis caused by persistently loose monetary policy, shadow banking and bank risk-taking channels have received widespread attention. There is a thought-provoking and nuanced relationship between central bank communications, shadow banking, and bank risk-taking [5].

Scholars were the first to consider how shadow banking affects the effectiveness of monetary policy. Banks’ existence of off-balance-sheet shadow banking complicates the transmission of monetary policy. The shadow bank is already in a black box, affecting the traditional transmission mechanisms such as monetary and credit channels of monetary policy [33, 34], impacting the traditional monetary multiplier theory and increasing the difficulty of economic regulation by monetary authorities [35, 36]. Of course, the fact that shadow banking interfered with the risk nonneutrality of banks before the 2008 financial crisis and played an important driving role in the overall risk-taking channel has led to skepticism [37]. After the crisis, the impact of shadow banking on bank risk-taking has been controversial but still valued [38, 39]. Some literature has examined shadow banking as a major factor and how it affects commercial banks’ risk-taking. There is also literature on shadow banking as a moderating factor of the impact of traditional monetary policy instruments on commercial bank risk-taking.

Existing studies have focused on the impact of traditional monetary policy tools on bank risk-taking. The study of the effectiveness of nonbank subjects of central bank communication and shadow banking and traditional channels of monetary policy, with significant academic contributions, is the logical starting point and theoretical basis of this paper. At the same time, few current studies include central bank communication, shadow banking, and bank risk-taking in the same logical and analytical framework for a detailed and in-depth discussion. This paper attempts to clarify the relationship between the three by conducting an in-depth study of the impact of central bank communication and shadow banking on bank risk-taking. Based on the M-S model of Morris et al. [32], we construct a novel model that includes central bank communication, shadow banking, and bank risk-taking. We propose hypotheses based on this model, measure the written communication index of central bank communication using the word extraction method, combine the data of 35 listed banks in China, and use the PVAR model to carry out an empirical test.

## 3. Economic model

Both central bank communication and shadow banking affect the risk-taking of commercial banks. For this reason, we draw on Keynes’s "beauty contest" idea, and based on the M-S model of the impact of public information on social welfare by Morris et al. [32], we introduce shadow banking to construct a new model. We theoretically explore the relationship between a central bank’s written communication, shadow banking, and banks’ risk-taking and propose corresponding research hypotheses based on the derivation results of the theoretical model.

First, consider all banks as a whole and assume that banks are uniformly distributed in the interval [0,1], *p*_*i*_ is the preference of bank *i* for risk, *p* is the preference of banks as a whole for risk, and $\overset{-}{p}\equiv \int_{0}^{1}p_{j}d_{j}$ is the average risk preference of banks as a whole. *Q*_*i*_ is the selection difference between bank *i* and bank *j*, and $Q_{i}\equiv \int_{0}^{1}{{\;(p}_{i}-p_{j})\;}^{2}dj,\;\overset{-}{Q_{i}}$ is the average selection difference of all banks, and $\overset{-}{Q_{i}}=\int_{0}^{1}Q_{j}dj$. υ is the basic economic state.

The utility of bank *i* is composed of two parts: the first is the distance between bank *i*’s risk appetite *p*_*i*_ and the underlying economic state υ, which is called "judgmental welfare loss"; and the second is the distance between bank *i*’s choice *Q*_*i*_ and bank’s average choice $\overset{-}{Q_{i}}$, which is called the "coordinated welfare loss". Banks *i* judge the weight of the two parts according to the externality, assuming that the weight of the coordinated welfare loss is λ, then the weight of the judged welfare loss part is 1 − λ, the larger λ the greater the externality, and the existence of externalities causes social inefficiency. At the same time, we assume that the externality is a zero-sum game, which means the gain for the winner is the cost for the loser.

Consequently, the utility function of bank *i* is expressed as follows.


$$u_{i}\left(p,v\right)=-\left(1-\lambda \right){\left(p_{i}-v\right)}^{2}-\lambda \left(Q_{i}-\overset{-}{Q}\right)$$ (1)

Clearly, the utility of the entire banking system is defined as the average of the (normalized) utility of each bank, denoted as

$$W\left(p,v\right)=\frac{1}{1-\lambda }\int_{0}^{1}u_{i}\left(p,v\right)d_{i}=-\int_{0}^{1}{\left(p_{i}-v\right)}^{2}d_{i}$$ (2)

The central bank is concerned with the utility of the entire banking system, so it will strive to make the actions of all individual banks as close as possible to the state of the entire economy υ.

On the other hand, bank *i* pursues its own utility maximizing first-order conditions, as reflected by

$${p_{i}}^{*}=\left(1-\lambda \right)E_{i}\left(\upsilon \right)+\lambda E_{i}\left(\overset{-}{p}\right)$$ (3)

where $\overset{-}{p}\equiv \int_{0}^{1}p_{j}d_{j}$, is the average risk appetite of the banking system, and *E*_*i*_(·) is the expectation of υ and $\overset{-}{p}\;$ by banks after obtaining the information communicated by the central bank.

If the underlying economic state υ is known, then for all banks, *p*_*i*_ = *υ*, utility is maximized at economic equilibrium, and there is no conflict between individual rationality and overall banking system optimality. The reality, however, is that the basic state of the economy in the intricate economic and financial environment υ is uncertain. Thus, we consider the case of economic state υ uncertainty. At this point, banks have access to public information since public information is available to all banks, denoted here by *y*.


y=υ+η (4)

When shadow banking is not considered, η is a bias in the central bank’s understanding of the underlying economic state. η obeys a normal distribution with variance $\sigma_{\eta }^{2}$ and the precision of the central bank communication is $\alpha =\frac{1}{\sigma_{\eta }^{2}}$.

In addition to the central bank’s public information, bank *i* also obtains private information, which is assumed not to be available to other banks, denoted by *x*_*i*_.

$$x_{i}=\upsilon +\xi_{i}$$ (5)

where *ξ*_*i*_ is independent of υ, η. When shadow banking effects are not considered, *ξ*_*i*_ obeys a normal distribution with variance $\sigma_{\xi_{i}}^{2}$ and private information precision *$\beta =\frac{1}{\sigma_{\xi_{i}}^{2}}$*.

We consider the existence of a shadow banking situation. The emergence of shadow banking in China stems from bank deposit-to-loan ratio restrictions. Like foreign shadow banks, they are innovative tools for off-balance-sheeting banks’ high-risk assets, which can lead to ever-increasing real leverage of the banking system. It is evident that while shadow banking helps banks pursue high profits, it also leads to a continuous accumulation of risks [40]. More importantly, multiple nested and structured shadow banking products hide much important information about the banking system and are extremely opaque. They are outside the supervision of the monetary authorities, and central banks have increased difficulties in capturing bank risks and the current state of the economy. It can be seen that the existence of shadow banks increases the difficulty of the central bank in grasping the basic economic state, resulting in a deviation of the central bank’s understanding of the basic economic state from η to *τ* and the variance increases from $\sigma_{\eta }^{2}$ to $\sigma_{\tau }^{2}$. Then, the accuracy of central bank communication becomes $\alpha_{s}=\frac{1}{\sigma_{\tau }^{2}}$, which is smaller than the communication accuracy $\alpha =\frac{1}{\sigma_{\eta }^{2}}$ when shadow banks are not considered. Here, assuming that *s* represents the shadow bank, $\alpha_{s}=\frac{1}{\sigma_{\tau }^{2}}=s\alpha =\frac{s}{\sigma_{\eta }^{2}},\;0<s<1$, and so the public information after considering the shadow bank is:

$$y_{s}=\upsilon +\tau$$ (6)

Due to Chinese banks’ unique historical background and institutional characteristics, they are susceptible to central bank information but reflect little in their own information communication. Meanwhile, Chinese shadow banking is essentially a shadow banking system and a linear extension of banks’ traditional lending business [41]. Therefore, we do not change the assumption of the private information of banks and argue that shadow banking happens to be a reasonable means for banks to respond to the central bank’s communication and regulation.

Based on the above analysis, the risk appetite $p_{i}^{*}(x_{i},y_{s})$ is chosen by bank *i* when faced with public information *y*_*s*_ = υ + *τ* and private information *x*_*i*_ = υ + *ξ*_*i*_. Therefore, the conditional expectation of bank *i* for the basic economic state υ and the private information of other bank *j* is expressed as

$$E_{i}(\upsilon |x_{i},y_{s})=E_{i}(x_{j}|x_{i},\;y_{s})=\;\frac{\beta x_{i}+\alpha_{s}y_{s}}{\alpha_{s}+\beta }$$ (7)

Drawing on Cornand and Heinemann [42], the economy as a whole is assumed to face a linear information function.


$$p_{i}x_{j},y_{s}=kx_{j}+\left(1-k\right)\;y_{s}$$ (8)

Then the optimal risk appetite of banks is

$$p_{i}x_{i},y_{s}=kx_{i}+\;\left(1-k\right)\;y_{s}$$ (9)

The bank’s estimate of the average optimal risk appetite of other banks is

$$E_{i}\left(\overset{-}{p}\right)=k\left(\frac{\alpha_{s}y_{s}+\beta x_{i}}{\alpha_{s}+\beta }\right)+\;(1-k)\;y_{s}$$ (10)

Substituting Eqs (7) and (10) into Eq (3), we obtain

$${p_{i}}^{*}\left(x_{i},y_{s}\right)=\left(1-\lambda \right)E_{i}\left(v\right)+\lambda E_{i}\left(\overset{-}{p}\right)$$

$$=\;\left(1-\lambda \right)\left(\frac{\alpha_{s}y_{s}+{\beta x}_{i}}{\alpha_{s}+\beta }\right)+\lambda \left[\left(\frac{k\beta }{\alpha_{s}+\beta }\right)x_{i}+\left(1-\frac{k\beta }{\alpha_{s}+\beta }\right)y_{s}\right]$$

$$=\beta \left(\frac{\lambda k+1-\lambda }{\alpha_{s}+\beta }\right)x_{i}+\left(1-\frac{\lambda k+1-\lambda }{\alpha_{s}+\beta }\beta \right)y_{s}$$ (11)

By comparing the coefficients of Eqs (9) and (11), we can see that

$$k=\frac{\beta \left(\lambda k+1-\lambda \right)}{\alpha_{s}+\beta }$$ (12)

Solving for this, we obtain

$$k=\frac{\beta (1-\lambda )}{\alpha_{s}+\beta \left(1-\lambda \right)}$$ (13)

Then the optimal risk appetite chosen by bank *i* is

$${p_{i}}^{*}\left(x_{i},y_{s}\right)=\phi \left(y_{s}\right)=\frac{\beta \left(1-\lambda \right)}{\alpha_{s}+\beta \left(1-\lambda \right)}x_{i}+\frac{\alpha_{s}}{\alpha_{s}+\beta \left(1-\lambda \right)}y_{s}$$ (14)

Derivation of the central bank communication information *y*_*s*_ in the presence of shadow banking yields

$$\frac{\partial \phi (y_{s})}{\partial y_{s}}=\frac{\alpha_{s}}{\alpha_{s}+\beta (1-\lambda )}>0$$ (15)

Based on the above theoretical model derivation, the following hypothesis is proposed.

**Hypothesis 1:** Central bank communication positively affects banks’ risk-taking preferences; the more adequate the central bank’s information communication, the greater banks’ risk-taking preferences.

Derivation of the shadow bank *s* in Eq (14) yields

$$\frac{\partial \phi \left(y_{s}\right)}{\partial s}=\frac{\beta \left(1-\lambda \right)\;(y_{s-}x_{i})\;}{{\left[\frac{s}{\sigma_{\eta }^{2}}+\beta \left(1-\lambda \right)\right]}^{2}\sigma_{\eta }^{2}}$$ (16)

Eq (16) shows that the relationship between bank risk-taking and shadow banking depends on the degree of public and private information saturation. The information conveyed by the central bank is of general interest and market participants extract useful information from it on which to base their decisions.

In the short term, it is often found in theory and reality that central banks do not grasp current economic conditions well [43]. The accuracy of central bank communication is influenced by a number of factors such as market conditions, national contingencies, specific dates or deadlines [44], and deliberate failure to inform. At the same time, there is ambiguity in central bank communications, with the pursuit of too many objectives leading to inconsistent communications and conflicting expressions in interperiod central bank communications. It is evident that imperfect information to assess the basis of central bank decisions or monetary policy can lead to complications in the private sector’s response to central bank policy [45]. There are forecasting and credit behavior frictions in banks’ communication of information to the central bank [46], and it is very difficult to determine the short-term transparency and information content of the central bank [47]. In other words, in the short term central bank communication cannot improve the accuracy of market participants’ forecasts. In contrast, commercial banks generally have a relatively greater grasp and saturation of their own information over a shorter period, especially in the early stages of carrying out shadow banking, where the technical means are relatively simple and commercial banks can control them more easily. Thus $\left(y_{s-}x_{i}\right)<0$, and

$$\frac{\partial \phi \left(y_{s}\right)}{\partial s}=\frac{\beta \left(1-\lambda \right)\;(y_{s-}x_{i})\;}{{\left[\frac{s}{\sigma_{\eta }^{2}}+\beta \left(1-\lambda \right)\right]}^{2}\sigma_{\eta }^{2}}<0$$ (17)

In the long run, forward-looking guidance on central bank communication has sustained predictive power [48]. Monetary policy indicators such as interest rates evolve along the path the central bank wishes [1]. Central bank communication indicators are three months ahead of economic conditions [12], and financial markets are vulnerable to central bank communication of long-term objectives [49]. Central bank communication can greatly influence monetary policy expectations, changing risk premia to shape long-term interest rates [50]. Central bank communication cannot reduce the divergence of expectations of market participants in the short term, but it is indeed very effective in the long term [44]. As a key player in the financial markets and a key hub for information collection and processing, banks will continue to digest and absorb central bank public information and endogenously adjust their future expectations of the path of monetary policy in light of new economic developments as external information becomes more certain.

Over a long period, the dynamics of banks’ business decisions and the overall situation will be more complex, especially as it will be difficult to fully and effectively capture the technical complexity and measurement difficulties of shadow banking. Through constant innovation and nesting, shadow banking has created an extraordinarily complex and difficult process for clarifying the structure, resulting in a large amount of idle funds. Coupled with the incentive to avoid regulation, shadow banking exploded [51]. The subprime crisis is a good example of the inability of commercial banks to grasp the information about themselves that underlies shadow banking. The existence of shadow banking makes it difficult to clearly access and measure individual information such as the efficiency and profitability of banks, the accumulation of risks in the financial system and information on deep individual environmental decisions such as the real economy and national financial stability [52]. This seriously reduces the transparency of banks’ balance sheets [53].

Therefore, in the long run, the external public information communicated by the central bank is more certain for the individual bank, while its own internal private information is less precise, so that $\left(y_{s-}x_{i}\right)>0$, when

$$\frac{\partial \phi \left(y_{s}\right)}{\partial s}=\frac{\beta \left(1-\lambda \right)\;(y_{s-}x_{i})\;}{{\left[\frac{s}{\sigma_{\eta }^{2}}+\beta \left(1-\lambda \right)\right]}^{2}\sigma_{\eta }^{2}}>0$$ (18)

Therefore, we propose the following hypothesis.

**Hypothesis 2**: The existence of shadow banking in the context of the release of public information by the central bank is beneficial to banks in the short run to reduce risk-taking appetite but may lead to higher risk-taking by banks in the long run.

Another point is that although some studies have argued that the monetary policy transmission mechanism is overall similar across countries [54], there is still variability. State-owned and large banks in developed economies are more closely related and more sensitive to monetary policy [55]. However, Chinese scholars have found that large state-owned banks’ (Bank of China, Agricultural Bank, Industrial and Commercial Bank, Construction Bank and Postal Reserve Bank) monetary policy transmission effects are not ideal [36]. Ma and Wang [56] attribute this phenomenon to the difference in financial systems. The dominant financial systems in developed countries such as the UK and the U.S. are the financial markets. In China, however, it is banks, while the regulatory system of the Chinese banking sector (deposit to loan ratio, legal reserve, and capital adequacy ratio) is strict and well established. As a result, the size of loans is subject to many restrictions, which may weaken the transmission effect of monetary policy.

In addition, when we consider the issue of banks in China, a point that cannot be circumvented is the size of banks and the nature of their property rights. In China, unlike small and medium-sized joint-stock banks, the more specific nature of state-controlled property rights results in large state-owned banks having the following characteristics. ① The pursuit of profit is less motivated and more focused on safe and sound operations. Unlike small and medium-sized joint-stock banks that constantly pursue profit maximization goals and relatively short-term appraisal mechanisms, large state-owned banks are not intrinsically driven by the pursuit of profit. Sound and safe operations are their bottom line, which determines bank leaders’ promotion, and relegation and career development [36]. Safety bottom-line thinking has led large state-owned banks to be relatively risk-averse and inclined to lower risk-taking. ② Banks implement national policies and are not sensitive enough to the information communicated by the central bank. Large state-owned banks have high asset and liability ratios, with funds flowing to enterprises of a state-owned nature. In particular, large state-owned enterprises’ capital use compliance and rigidity make large state-owned commercial banks insensitive to loose policies and central bank communication. However, small and medium-sized banks have relatively low gearing ratios, and their intrinsic profit motive drive also requires them to expand their business scale. Private enterprises are the main subjects of their fund utilization. Based on relatively single domestic financing channels, small and medium-sized banks have more substantial bargaining power over private enterprises. However, at the same time, they must pay close attention to the policy guidance of the monetary authorities. In other words, they are more sensitive to central bank communication and exchange. ③ The banking system is in an important position, and the central bank’s communication has given more consideration to the stakes. Large state-owned banks are highly susceptible to risk transmission to the entire economic system as they are systemically important banks. PBOC communication has considered the policy’s possible impact on them. Nevertheless, systemic risks are unlikely to arise for city and rural banks. Small and medium-sized joint-stock banks’ communication functions are less considered by small and medium-sized joint-stock banks, which may have a greater impact on risk-taking than the central bank’s communication. Limited operating areas, relatively fixed sources of funding, and limited ability to place funds, coupled with rigid deposit-to-loan ratio regulation, force small and medium-sized joint-stock banks to be more proactive in shadow banking [36, 57], all of which cause a rise in bank risk-taking and greater sensitivity to central bank communication.

**Hypothesis 3:** The impact of central bank communication on the risk-taking of small and medium-sized joint-stock banks is more pronounced in shadow banking than large state-owned banks.

## 4. Empirical model and data

### 4.1. Empirical design

In the literature, VAR models have been widely used in studying monetary policy transmission channels [58], and the research involved in the risk-taking channel is no exception [59]. Although VAR models are useful for addressing the linkages between monetary policy and economic agents and do not rely on economic theory as much as structural equation models do, they suffer from the "curse of dimensionality", meaning they cannot be applied to panel data. Therefore, some scholars have expanded their thinking and introduced the panel PVAR model into the research of bank risk-taking [60]. Considering the research topic of this paper and the fact that the PVAR model is very suitable for short time series, this paper uses this model for empirical testing.

Based on the study, we developed the following main regression model.

$$Y_{it}=a_{it}+\sum_{j=1}^{p}A_{j}Y_{i,t-j}+d_{t}+f_{i}+\varepsilon_{it}$$ (19)

i∈{1,2,……,N},t∈{1,2,……,T}

where *Y*_*it*_ is a 1 × *k* dimensional vector of five variables at year *t* of the *i*-th bank, expanded as follows:

Yit=EPEtSBtCItBRTitRISKit (20)

where *EPE* represents macroeconomic conditions, policy environment (interest rates, economic conditions, price level), *S*.*B*. represents shadow banking size variables, *CI* represents central bank communication index, *BRT* represents bank-level factors, *RISK* represents bank risk-taking, *a*_*it*_ represents the longitudinal intercept, A_j_ is the parameter matrix, *j* represents the lag order of the variable, *d*_*t*_ is the time effect vector of the PVAR model, *f*_*i*_ is the individual fixed effect of the PVAR model, and *ε*_*it*_ represents the random disturbance term of the model. This variable order draws on Buch, Eickmeier and Prieto [61] and Zhao and He [60], where macro variables are placed before micro variables. For the order of the two variables of shadow banking and central bank communication, a robustness test of randomly changing the order follows.

### 4.2. Variables and data description

#### 4.2.1. Building a central bank communication index

Central bank communication is how the central bank announces information on monetary policy objectives, intermediary indicators, operational indicators, and monetary policy tools to market participants. The literature often uses the wording extraction method in document analysis to extract, filter, and integrate critical statements from some public reports of the central bank as indicators of central bank communication. Because the forms of written communication such as the "Monetary Policy Implementation Report" and the "Resolution of the Monetary Policy Committee Meeting" are relatively fixed and more formal and authoritative, they have become the main objects of the word extraction method. Moreover, the word extraction method is objective and systematic, so this paper also does the same. We mainly adopt the word extraction method for the "China Monetary Policy Implementation Report" (hereinafter referred to as the "Report"). We draw on the methods used by Heinemann and Ullrich [13], Bian and Zhang [62], and Gao and He [29] to construct the central bank’s communication index (*cinei*, hereinafter referred to as the central bank communication index) for the domestic macroeconomic and policy situation as a proxy variable for central bank communication. This paper considers that China’s commercial banks occupy an important position in the world. According to the 2022 "The Banker" Global Banking Brand Value Ranking, 20 banks in China have entered the world’s top 100 banks, and 48 banks have entered the world’s list of the top 500 banks. In addition, the business scope of Chinese commercial banks is transnationalized and globalized, and their risk-taking is closely related to the global economic situation and policy situation. Therefore, this paper innovatively constructs the central bank’s communication index (*ciwai*), on foreign macroeconomic and policy conditions as a robustness test variable for central bank communication.

The People’s Bank of China (PBOC) has been publishing the Report on the PBOC official website quarterly since 2001, which provides an in-depth analysis of the macroeconomic and financial situation, explains monetary policy operations, and discloses the next monetary policy orientation. It is the core channel of communication between the PBOC and market players. In order to avoid the problem of duplication, we only select the text part for wording statistics. In order to ensure the validity of the statistics, we exclude the extreme cases that may be caused by major black swan events, such as the financial crisis in 2008 and coronavirus epidemic in 2020. The People’s Bank of China formally introduced "expectation management" in 2009, so we selected 44 issues from the first quarter of 2009 to the fourth quarter of 2019. The text of the Report is used as the basis to construct the central bank’s communication index using the following.

We extracted keywording from the Report. From the 44 issues of the Report, the People’s Bank of China (PBOC) selected the key terms of the domestic and foreign policy environment (easing, moderate easing, steady, tightening, and tightening), economic development (overheating, rising, stable, falling and recession), price trend (inflation risk, rising, stable, falling and deflation), liquidity situation (excess, abundant, moderate, insufficient and shrinking), employment level (high, good, moderate, falling and low) and real estate market situation (overheating, rising, stable, falling and recession). The frequency of each phrase and related descriptions in each issue of the Report (see Table 1) is counted as the raw data basis for constructing the central bank’s domestic (*cinei*) and foreign (*ciwai*) communication index.

**Table 1:** Specific expressions of the wording extracted from the monetary policy implementation report.

Phrase Type	Subdivision	Assignment value	Detailed Expression
Policy Environment	Easing policy	1	Easing Policy. Active Fiscal Policy, Investment Policy, Expanding Domestic Demand, Growth, Capital Support Policy, Foreign Trade Policy, Tax Reduction, Policy Revenue Reduction, Stimulus (Consumption) Policy, Financial Policy Support
	Moderate easing policy	0.5	Moderate, Reasonably Lenient, Controlled
	Stable Policy	0	Robust, Neutral, Moderate
	Moderate Austerity	-0.5	Tightening And Tightening of Policies
	Austerity	-1	Moderate Tightening
Macroeconomic conditions	Economic overheating	1	Overheated and Fast-Growing Economy
	Economic Growth	0.5	Fast Economic Growth
			Stable (Restorative) Economic Growth, Rebound, Steady Development, Positive Economic Situation, Improving Fundamentals
			Economic (Heat) Expectations Index Up, Confidence Up
	Economic Stability	0	Stable, Healthy, Orderly, Recovery, Stabilization, Easing
	Economic decline	-0.5	Cold Economy, Gloomy Outlook
			Economic Growth Decelerates, Declines, Slumps, Slows
	Recession	-1	Deterioration, Recession, Weakness
Price level	Inflation Risk	1	Excessive Price Increases, Inflation Risk
	Price increases	0.5	Inflationary Pressure, Upward (Rising) Price Pressure
	Price stability	0	Stable, Stable Prices
	Decrease in prices	-0.5	Falling Prices, Downward Pressure (CPI, PPI, GDP Deflator Or Deflator, Commodities). Lower Prices. Deflationary Pressure, Lower Inflation
	Deflation	-1	Deflation
Liquidity level	Excess liquidity	1	Excess Liquidity
	Enhanced liquidity	0.5	Ample or Enhanced Liquidity, Increased (Injected) Liquidity; Moderately Accommodative Liquidity, Generally Accommodative Liquidity, Generous, Persistently Accommodative
	Moderate liquidity	0	Moderate, Reasonable Liquidity
	Lack of liquidity	-0.5	Insufficient Level of Liquidity
	Shrinking liquidity	-1	Liquidity Levels Shrink
Full employment level	High level of employment	1	High Employment Rate, Low Unemployment Rate
	Increased employment levels	0.5	Unemployment Pressure Eases, Overall Employment Level is Good, Employment Growth, Capacity Increases, Employment Status Index Improves and Increases
			Employment (Situation) is Expected to Improve
	Moderate employment level	0	Stable Employment, Reasonable Employment Levels
	Lower employment levels	-0.5	decline In Employment, Increase in Unemployment
	Very low level of employment	-1	High Pressure on Employment and Unemployment; Deteriorating Employment Situation (Not Optimistic); Unemployment Rate at Historically High
Real Estate Market Status	Housing market overheating	1	Overheated, Surging, Persistently High, Rising Too Fast
	Rising housing market	0.5	Growth in Commercial Property Sales, Rebound, Improvement, Rapid Sales Growth, Active Real Estate Market Transactions
			Home Sales Price Increases (Year-on-Year, Month-on-Month)
			Increase in Commercial Real Estate Loan Balances
			New Balance of Real Estate Development Loans
			Home Purchase Loans, Personal Housing Loans Added
			Increase in Real Estate Loans, Increase in The Proportion of Real Estate Development Investment (Year-on-Year, Month-on-Month)
	Stable housing market	0	Stabilize
	Falling housing market	-0.5	Sales of Commercial Properties Fall, Real Estate Market Continues to Decline
			Home Sales Price Decline (Year-on-Year, Month-on-Month)
			Decrease in Commercial Real Estate Loan Balances
			Decrease in Balance of Real Estate Development Loans
			Decrease in Home Purchase Loans
			Lower Proportion of Real Estate Development Investment (Year-on-Year, Month-on-Month)
	Housing Market Recession	-1	Depressed, Under Pressure, No Improvement, Continued Decline

Note: Description of the assigned values. Drawing on Boschen and Mills [63] and Wang et al. [26], each of the above types of wording was divided into five magnitudes and assigned weights of 1, 0.5, 0, -0.5, and -1 according to wording strength and direction, respectively.

We construct a central bank communication index based on an a priori formula. Based on the information shown in Table 1, the study constructs the central bank disclosure index Formula (14) according to Heinemann and Ullrich [13] and Wang et al. [26], and the statistical frequencies were weighted and summed. The details are as follows.

$$CI_{t}=\sum_{i=1}^{n}\frac{nobs\;\left(x_{it}\right)-meannobs\;\left(x_{i}\right)\;}{std\;\left(x_{i}\right)\;}\alpha_{i}$$ (21)

where *CI*_*t*_ is the central bank communication index in period *t*, which is the result of standardizing the effective wording *x*_*i*_, *nobs* (*x*_*it*_) is the word frequency of *x*_*i*_ in period t of the Report, *meannobs* (*x*_*i*_) is the average word frequency of *x*_*i*_ in each period of the Report, *std* (*x*_*i*_) is the standard deviation of the word frequency of *x*_*i*_ in each period of the Report, and *α*_*i*_ is the weight of effective wording *x*_*i*_, which is obtained by calculating the percentage of deviation. The final constructed central bank communication index *CI*_*t*_ (*cinei*_*t*_ and *ciwai*_*t*_ are calculated separately, but the method is exactly the same) is proportional to the easing message of the central bank communication. The larger the index is, the more easing the central bank communication regulation is in that period.

#### 4.2.2. Shadow banking indicators

Most of the literature selects its size indicator for the empirical study of Chinese shadow banking. The current methods used by scholars to measure the size of shadow banking mainly include macro estimation by the backward projection of shadow banking financing according to an estimated ratio of GDP or summing up the estimated size of each component of shadow banking, and the method of replacing the whole with the part by summing up the size of the main operations of undiscounted bank acceptance bills, entrusted loans, and trust loans [64, 65]. The macro estimation method has strong subjectivity and poor accuracy, and the estimation aggregation method has problems with double-counting, long intervals, and low data frequency. Therefore, combined with the comprehensive consideration of the research content and at the same time to avoid overestimating of the shadow banking scale, we use the method of partial instead of whole. The method is adopted by most of the literature to calculate the growth rate of undiscounted bank acceptance bills, entrusted loans, and trust loan scale as shadow banking proxy variables (*sbschr*).

#### 4.2.3. Bank risk-taking indicators

The current selection of bank risk-taking variables has two main perspectives: active and passive. First, the active risk-taking measures include the risk-weighted asset ratio [66, 67], nondeposit liabilities ratio [59, 68], expected default rate [28], and bank lending standards [11, 12, 61]. Second, the passive risk-taking measures are the nonperforming loan ratio [60, 66], Z-value [8, 35, 39, 69], and loan loss provisioning ratio. Based on previous experience and combined with the novel theory, we select the proportion of nondeposit liabilities (*rndd*) as a proxy variable for banks’ (active, liability-side) risk-taking based on a comprehensive consideration of multidimensional bank risk-taking. The nonperforming loan ratio (*nlr*), which can reflect banks’ passive, asset-side risk-taking, is taken as a robustness test indicator.

#### 4.2.4. Bank-level factor indicators

Referring to existing studies [6, 68, 69], we select banks’ capital adequacy ratio (*car*) as a microlevel proxy variable.

#### 4.2.5. Macroeconomic conditions and policy environment indicators

According to Maddaloni and Peydro [6] and Xu and Chen [69], the effect of the duration of the accommodative monetary policy variable (*plmp*) is incorporated by running an OLS regression of the annual benchmark interest rate on loans on the growth rate of the economy (*gdp*) and the growth rate of the price level (*cpi*) to obtain Taylor residuals (*tr*), with positive residuals representing a tight monetary policy environment (relatively high monetary policy rate), negative residuals representing an accommodative monetary policy environment (relatively low policy rate), and the duration of accommodative money (*plmp*) being the number of years in which the residuals are continuously negative.

The data sources and descriptions of the main variables are shown in Table 2. The sample dataset is from 2009 to 2019. For the very few missing data points, the mean values of the adjacent two years were taken to check and fill. Then balanced panel data were generated and completed by STATA system operation.

**Table 2:** Specific description of the main variables and data sources.

Variable Name	Specific instructions	Data source
Central Bank Communication Index (*cinei*, *ciwai)*	Monetary Policy Implementation Report wording extraction, calculation construction	Author measurement
Shadow banking scale growth rate (*sbschr)*	The sum of the growth rate of entrusted loans, trust loans and undiscounted bank acceptance bills	WIND
Percentage of non-deposit liabilities (*rndd)*	Percentage of non-deposit liabilities = non-deposit liabilities/total assets	WIND
Non-performing loan ratio (*nlr)*	Non-performing loan ratio = Non-performing loans/total loans	WIND
Bank Capital Adequacy Ratio (*car)*	Capital adequacy ratio = net capital / risk-weighted assets	WIND
Duration of accommodative monetary policy (*plmp)*	Regressing interest rates on economic growth and *cpi*, the residuals of which are Taylor’s rule residuals, using Taylor’s rule residuals less than zero for the duration	Author measurement

### 4.3. Statistical characteristics of variables and data smoothness test

#### 4.3.1. Descriptive statistics of each variable

Descriptive statistics for the main proxy variables are presented in Table 3. The mean value of the central bank communication index (*cinei*) is -0.080, with a maximum value of 6.690 and a minimum value of -6.090, indicating that the attitudes and intentions reflected in central bank communication vary considerably from period to period. The average growth rate of the shadow banking scale is -3.12%, the maximum value is 126.5%, and the minimum value is -196%, mainly because after the financial crisis in 2009, China introduced a series of new regulations and policies on shadow banking to control the scale of shadow banking. The guiding effect of preventing systemic risk is pronounced, and the growth rate of China’s shadow banking scale is on a downward trend after 2016. The banks’ risk-taking proxy variable nondeposit liability ratio (*rndd*) has a mean value of 23.78%, a maximum value of 49.9%, and a minimum value of 0. Meanwhile, the nonperforming loan ratio (*nlr*) has a mean value of 1.230%, a maximum value of 3.88%, and a minimum value of 0.16%, indicating that risk-taking varies widely across banks. The average value of the duration of accommodative monetary policy (*plmp*) is 1.182, with a maximum value of 4 and a minimum value of 0. This indicates that China’s central bank adopts a monetary policy with varying degrees of accommodative tightness in different periods of economic development, and the duration and the width of adjustment are larger. The capital adequacy ratio (*car*) has a mean value of 13.14%, a maximum value of 40.3%, and a minimum value of 8.33%, which are in line with reality.

**Table 3:** Descriptive statistics of each variable.

Variables	N	Mean	Sd	Max	Min	P50	P25	P75
*cinei*	385	-0.080	3.190	6.690	-6.090	-0.030	-2.430	1.820
*ciwai*	385	-0.950	2.300	2.450	-4.870	-0.830	-2.550	1.040
*sbschr*	385	-3.120	88.06	126.5	-196.0	-6.690	-47.26	63.45
*rndd*	385	23.78	10.42	49.90	0	23.45	16.70	31.58
*nlr*	385	1.230	0.520	3.880	0.160	1.190	0.860	1.520
*car*	385	13.14	2.440	40.30	8.330	12.97	11.87	14.10
*plmp*	396	1.182	1.338	4	0	1	0	2

#### 4.3.2. Data stability test

To avoid spurious regression in the PVAR model, a unit root test of the variables is needed. The common LLC and IPS tests are selected here, and the results are presented in Table 4, where *d_plmp* is the unit root test of the variable *plmp* after the first-order difference, and the P value are all significantly 0 at the 1% level. The P value of the unit root tests of the original data of the other variables all reject the original hypothesis of the existence of a unit root at the 1% level, indicating that each panel variable is smooth and satisfies the requirements of further tests.

**Table 4:** Results of the unit root test of variables.

Variables	LLC			IPS		
	T-value	P-value	Stability	Statistic	P-value	Stability
*cinei*	-30.1755	0.0000	Stable	-21.3200	0.0000	Stable
*ciwai*	-9.9520	0.0000	Stable	-3.3533	0.0004	Stable
*sbschr*	-18.7281	0.0000	Stable	-13.6004	0.0000	Stable
*rndd*	-8.4201	0.0000	Stable	-2.5179	0.0059	Stable
*nlr*	-8.7730	0.0000	Stable	-3.6866	0.0001	Stable
*car*	-7.6025	0.0000	Stable	-3.5868	0.0002	Stable
*d_plmp*	-10.4802	0.0000	Stable	-4.7454	0.0000	Stable

## 5. Empirical tests and analysis of results

### 5.1. Central bank communication, shadow banking and bank risk-taking test results

Prior to the PVAR model analysis, this study first determined the optimal lag order to be 1 based on the AIC, BIC and HQIC. Stability tests are then carried out to demonstrate that the system of variables constructed in this paper was stable. The correlation between the variables is again confirmed by Granger causality tests, which find that the central bank communication index (*cinei*), the growth rate of shadow banking size (*sbschr*) and the duration of accommodative monetary policy (*d_plmp*) are Granger causes of active bank risk-taking (*rndd*) but not the capital adequacy ratio (*car*). The central bank communication index (*cinei*) and the duration of accommodative monetary policy (*d_plmp*) are causally related to each other. The absence of Granger causality between the central bank communication index (*cinei*) and the size of shadow banking (*sbschr*) supports, to some extent, the theoretical model’s assumption that shadow banking information is relatively hidden and that it becomes more difficult for central banks to understand and grasp the underlying economic state. Considering that both are again closely related to active risk taking by banks (*rndd*) as previously confirmed, this further proves the significance and feasibility of this paper’s research.

#### 5.1.1. GMM results for the PVAR model

To facilitate the distinction between subsequent empirical models, the main regression model is defined in this study as Model (1). The PVAR model uses the GMM approach to estimate the parameters, with all variables lagged by one period, and the estimation results are presented in Table 5. The lagged one-period domestic central bank communication index (*cinei*) is significantly positively correlated with banks’ active risk-taking (*rndd*) at the 10% level with a coefficient of 0.4175, indicating that the higher the degree of written communication from the central bank on easing policy, the greater the risk-taking of banks, thus confirming that Hypothesis 1 holds. Meanwhile, the one-period lagged growth rate of shadow banking size (*sbschr*) is negatively correlated with bank risk-taking (*rndd*) at the 5% significance level with a coefficient of -0.0054, which verifies Hypothesis 2 that shadow banking negatively affects bank risk-taking in the short run, but exactly what effect it will have in the long-run must be analyzed in conjunction with the subsequent impulse response plots.

**Table 5:** Model (1) PVAR model estimation results.

	*d_plmp*	*sbschr*	*cinei*	*car*	*rndd*
*d_plmp*(L1)	-0.6540***	-10.9100	1.0000***	0.3329***	1.6902***
	(0.0939)	(13.7725)	(0.2548)	(0.0917)	(0.5491)
*sbschr*(L1)	-0.0002	-0.3384***	0.0011	-0.0003	-0.0054**
	(0.0005)	(0.0661)	(0.0013)	(0.0006)	(0.0022)
*cinei*(L1)	-0.4081***	-5.1113	0.5493***	-0.0476	0.4175*
	(0.0323)	(5.5399)	(0.0909)	(0.0438)	(0.2266)
*car*(L1)	0.1156	-60.9159**	-0.8929**	0.5209***	-1.2436
	(0.0913)	(27.6741)	(0.3845)	(0.1145)	(0.7554)
*rndd*(L1)	0.1369***	-11.4864***	-0.3876***	0.0595***	0.4175***
	(0.0174)	(3.9454)	(0.0590)	(0.0193)	(0.1180)

#### 5.1.2. Impulse response analysis

**Fig 1:**
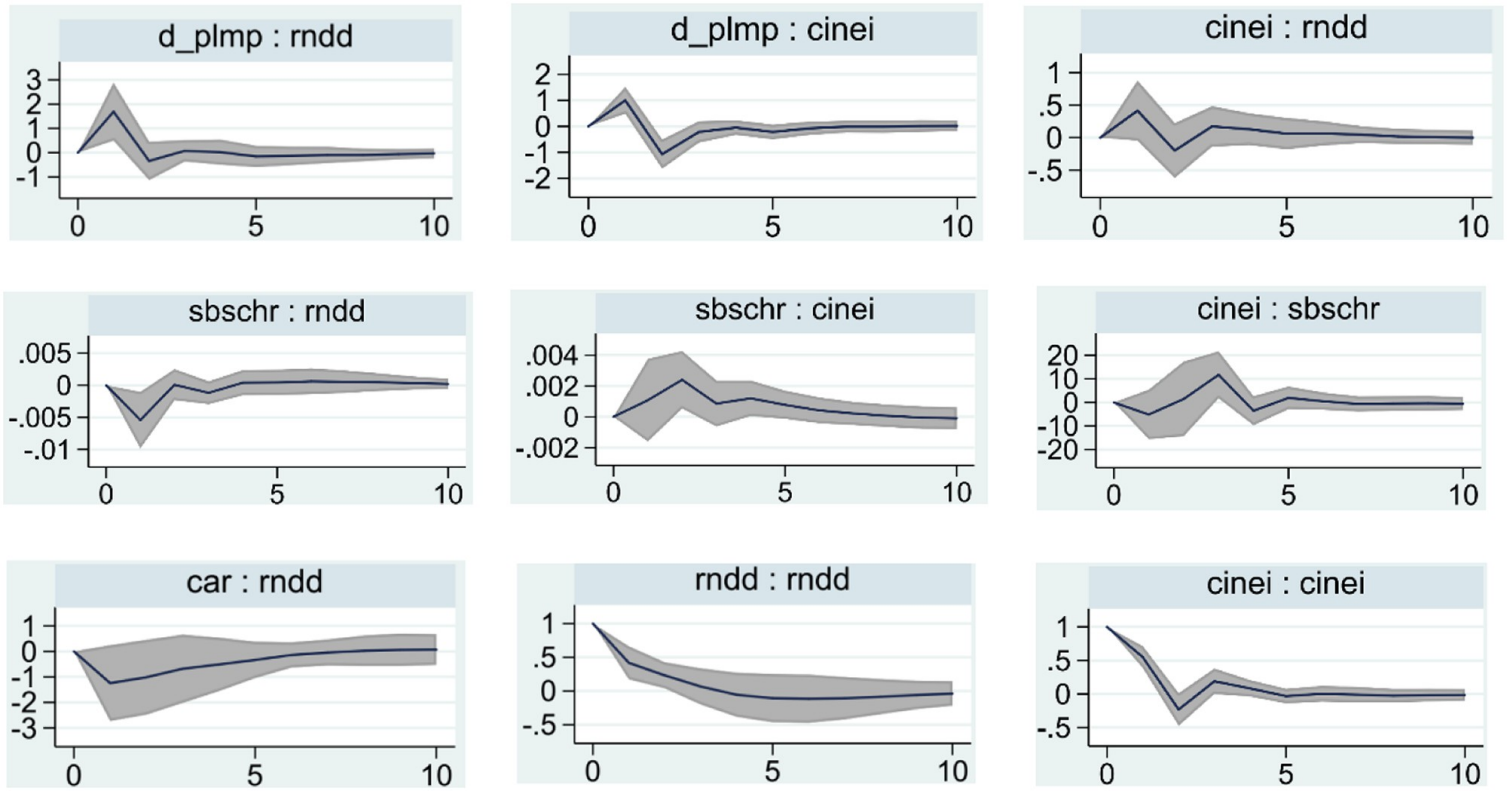
Model (1) 200 Monte-Carlo shock response graph.

Fig 1 presents a plot of the effects of 200 Monte-Carlo shocks between the five variables of interest in this paper: duration of accommodative monetary policy, shadow banking, central bank communication index, bank capital adequacy, and bank risk-taking. The number of lags is 10, and the top of each subplot is labeled "impulse: response" (impulse variable: response variable). The solid line is the 50% quantile level, and the gray part is the confidence interval at the 95% level. Based on the impulse response diagram, the following specific analysis is made.

There are existing tests of the monetary policy risk-taking channel in China (*d_plmp—rndd*). The first panel of the first row of Fig 1 shows that a one-unit shock to the duration of monetary policy (*d_plmp*) induces a 1.6902-unit isotropic change in bank risk-taking (*rndd*), which is lagged by one period and reaches a maximum in the first period, then turns weakly negative in the second period, but approaches zero and stabilizes in the third period and beyond. It is evident that an accommodative monetary policy environment will incentivize banks to increase their risk-taking. The bank risk-taking channel is actually more concerned with the impact of a sustained accommodative monetary policy environment rather than a short-term environment [5]. This point has long been easily overlooked, and this paper demonstrates that a channel for monetary policy risk-taking also exists in China from a perspective other than purely the policy interest rate or money supply [67, 69, 70].

There is evidence that accommodative monetary policy affects central bank communication and exchange (*d_plmp—cinei*). According to the second graph in the first row of Fig 1, it can be seen that a unit shock to the duration of easing monetary policy (*d_plmp*) can cause a positive response of approximately 1 unit to the central bank communication index (*cinei*) in the first period, and this positive relationship changes to a negative relationship in the second period, and stabilizes in the third and fourth periods close to 0. This shows that easing monetary policy positively affects the central bank’s communication on the easing economy and policy in the short term. The degree is pronounced, but with the growth of the duration of the easing monetary policy, the impact on the subsequent central bank communication index decreases or even becomes negative. With the increase in the implementation of the easing monetary policy time, the macroeconomic situation improves. Based on the principle of camera choice, central bank communication and communication will gradually become moderate, robust, or even tight communication.

Hypothesis 1 is evidence that central bank communication and exchange on accommodative economic policies positively affect bank risk-taking when shadow banking exists (*cinei—rndd*). According to the third panel in the first row, a positive shock to the central bank’s written communication index (*cinei*) causes a positive response of 0.4175 units in bank risk-taking (*rndd*) in the first period, after which this positive relationship slowly weakens to a negative response of approximately 0.2 units maximum in the second period, but then returns to a positive response in the third period, and maintains this stable positive relationship in the following periods until the eighth period when it gradually returns to near 0. This shows that the impact of central bank communication (*cinei*) on bank risk taking (*rndd*) is higher. In the short run, this effect is more volatile and more pronounced. Overall, a positive relationship can be maintained between the two. This is because central bank communication provides more information about the future, reduces uncertainty about policy and economic conditions, and increases the accuracy of commercial banks’ information forecasts. Central bank communication releases a sense of commitment from the central bank to the future. In the "odyssey" model of forwarding guidance (where central bank communication is a commitment), this feeling is amplified, and the effectiveness of central bank communication is enhanced [71]. Therefore, the increase in the strength and commitment of central bank communication to easing encourages banks to take higher risks; thus the empirical evidence supports Hypothesis 1.

We combine the above two parts of the analysis. It can be seen that "monetary policy continues to be eased—central bank communication index increases—bank risk-taking increases". This suggests that the central bank communication and exchange mechanism of the bank risk-taking channel exists in China. The easing monetary policy encourages banks to take higher risks by causing an increase in central bank communication.

Hypothesis 2 shows that shadow banking in the context of central bank communication affects bank risk-taking (*sbschr-rndd*). In the short run, shadow banking (*sbschr*) favors lower bank risk-taking (*rndd*), but in the long run shadow banking (*sbschr*) weakly and positively affects bank risk-taking (*rndd*). Looking at the first panel of the second row of Fig 1, a one-unit positive shock to the growth rate of shadow banking size (*sbschr*) will cause a decline in bank risk-taking (*rndd*), which is lagged by one period and produces a maximum negative impact of approximately 0.0054 units after one year. Then this negative impact shrinks and becomes a small positive impact and remains stable in the fourth period and beyond. Obviously, this is in line with the characteristics of the operation mode of shadow banking in reality and the interdependent relationship with banks. To circumvent the regulation, commercial banks can use shadow banking innovation as an immediate response. It achieves off-balance-sheet funding through channel business, interbank business, and nested business. The asset and liability position of commercial banks can be improved. Especially in the short term, shadow banking can help commercial banks offset the impact of adverse monetary policy changes and reduce their risk-taking. However, in the long run, shadow banking without supervision often has unregulated operations and relatively higher risks in investment and financing. It is embedded in many financial intermediaries and financial instruments, making the entire system is relatively fragile. When the macro environment is good, the cash flow is stable, there are no special shocks, and the risks are not exposed, the impact on the risk-taking of commercial banks is small. However, in a special event, its apparent separation and close relationship with commercial banks makes it difficult to effectively separate risks and is highly contagious, which leads to an increase in risk-taking of commercial banks and an increase in the growth rate of shadow banking.

There is a dynamic relationship between shadow banking and central bank communication. According to the second panel of the second row of Fig 1, a one-unit positive shock to the growth rate of shadow banking size (*sbschr*) causes an anisotropic response of 0.0011 units in the central bank communication index (*cinei*). It reaches a maximum of approximately 0.0025 units in the second period, finally declining gradually to approach zero in the eighth period and stabilizing. In general, to prevent the risk of the shadow banking system, the central bank’s communication and exchange of monetary policy should be tightened when shadow banks expand. This deviation may be because the shadow banks that avoid regulation increase the difficulty and deviation of the central bank in grasping the financial system and the basic economic state. According to the third panel in the second row of Fig 1, a one-unit positive shock to the central bank communication index (*cinei*) causes a response of -5.1113 units in the growth rate of shadow banking size (*sbschr*) in the first period. This negative response turns positive in the second period and reaches a maximum in the third period before gradually returning to zero in the sixth period. The above two sections show a long and complex dynamic relationship between shadow banking and central bank communication.

A bank’s capital adequacy negatively affects its risk-taking (*car—rndd*). According to the first panel in the third row of Fig 1, a one-unit shock to bank capital adequacy (*car*) causes a negative response in bank risk-taking (*rndd*). This negative relationship is lagged by one period, reaching a maximum in the third period and then gradually weakening, approaching zero and stabilizing in the seventh period. This is clearly in line with the majority of the literature on bank heterogeneity, which concludes that banks with higher capital adequacy ratios have lower levels of risk-taking.

#### 5.1.3. Analysis of variance decomposition

According to the impulse response diagram, it is easy to see an up-and-down relationship between central bank communication, shadow banking, and bank risk-taking, which is not linear. The connection between the three is still somewhat complex. The specific variance decomposition results are shown in Table 6. The variance decomposition results in periods 9 and 10 are consistent, which means that the system is stable after nine prediction periods.

**Table 6:** Model (1) variance decomposition results.

Step	Response Variable	Impulse Variable					Response Variable	Impulse Variable				
		d *plmp*	*sbschr*	*cinei*	*car*	*rndd*		d *plmp*	*sbschr*	*cinei*	*car*	*rndd*
1	d *plmp*	1	0	0	0	0	*car*	0.0424	0.0857	0.0191	0.853	0
2	d *plmp*	0.655	0.0164	0.0471	0.000970	0.281	*car*	0.175	0.0631	0.0138	0.712	0.0362
3	d *plmp*	0.480	0.0495	0.0750	0.00765	0.388	*car*	0.215	0.0492	0.0112	0.577	0.148
4	d *plmp*	0.448	0.0593	0.0732	0.0126	0.407	*car*	0.213	0.0485	0.0146	0.477	0.247
5	d *plmp*	0.426	0.0606	0.0705	0.0183	0.425	*car*	0.208	0.0533	0.0185	0.423	0.298
6	d *plmp*	0.413	0.0651	0.0722	0.0212	0.429	*car*	0.201	0.0571	0.0209	0.398	0.323
7	d *plmp*	0.408	0.0668	0.0726	0.0247	0.428	*car*	0.196	0.0605	0.0230	0.387	0.334
8	d *plmp*	0.407	0.0671	0.0726	0.0266	0.427	*car*	0.193	0.0624	0.0241	0.385	0.336
9	d *plmp*	0.407	0.0672	0.0726	0.0273	0.426	*car*	0.192	0.0632	0.0245	0.384	0.336
10	d *plmp*	0.407	0.0672	0.0725	0.0276	0.426	*car*	0.192	0.0634	0.0246	0.385	0.335
1	*sbschr*	0.157	0.843	0	0	0	*rndd*	3.18e-05	0.271	0.0767	0.0129	0.640
2	*sbschr*	0.116	0.742	0.00433	0.0764	0.0612	*rndd*	0.0140	0.244	0.0995	0.0710	0.572
3	*sbschr*	0.153	0.704	0.00477	0.0736	0.0646	*rndd*	0.0134	0.240	0.0941	0.0994	0.554
4	*sbschr*	0.149	0.681	0.00459	0.0711	0.0938	*rndd*	0.0159	0.238	0.0953	0.110	0.541
5	*sbschr*	0.149	0.667	0.00797	0.0697	0.106	*rndd*	0.0191	0.236	0.0945	0.114	0.536
6	*sbschr*	0.148	0.662	0.00840	0.0700	0.112	*rndd*	0.0219	0.234	0.0937	0.115	0.536
7	*sbschr*	0.147	0.659	0.00877	0.0706	0.114	*rndd*	0.0241	0.232	0.0930	0.114	0.537
8	*sbschr*	0.147	0.658	0.00902	0.0710	0.115	*rndd*	0.0250	0.231	0.0926	0.113	0.538
9	*sbschr*	0.147	0.658	0.00908	0.0714	0.115	*rndd*	0.0254	0.230	0.0925	0.113	0.539
10	*sbschr*	0.147	0.658	0.00909	0.0715	0.115	*rndd*	0.0255	0.230	0.0925	0.113	0.539
1	*cinei*	0.615	0.208	0.177	0	0						
2	*cinei*	0.418	0.133	0.115	0.0471	0.287						
3	*cinei*	0.360	0.129	0.119	0.0406	0.352						
4	*cinei*	0.345	0.123	0.107	0.0401	0.385						
5	*cinei*	0.326	0.121	0.103	0.0406	0.409						
6	*cinei*	0.315	0.123	0.104	0.0426	0.415						
7	*cinei*	0.311	0.124	0.103	0.0458	0.415						
8	*cinei*	0.310	0.125	0.103	0.0476	0.415						
9	*cinei*	0.309	0.125	0.103	0.0485	0.414						
10	*cinei*	0.309	0.125	0.103	0.0489	0.414						

Analyzing the variance decomposition results of banks’ risk-taking is the main concern of this paper. ① Banks’ risk volatility mainly originates from themselves, and although their contribution decreases from 64% in the first period to a stable 53.9% in the ninth period and beyond, their own contribution still accounts for the vast majority. ② The contribution of the positive effect of central bank communication on banks’ risk-taking increases from 7.67% in the first period to a maximum of 9.95% in the second period, then gradually to 9.25% in the ninth period and stabilizes. Yang and Zhou [72] argue that a variance decomposition contribution greater than 5% would indicate a causal relationship between the variables and is significantly valid, showing that the central bank communication index does significantly affect the risk-taking of banks, again proving Hypothesis 1 that central bank communication and communication positively affect the risk-taking of banks. ③ The contribution of shadow banking to banks’ risk-taking decreases from 27.1% in the first period to 24.4% in the second period relatively quickly, and then slowly decreases to 23% in the ninth period, which is the second-largest contributing factor to banks’ risk-taking itself, indicating that the impact of shadow banking on banks’ risk-taking is not only great in the short term but also very significant in the long term. This is consistent with short-term banks tending to engage in risky businesses that avoid regulation with the help of shadow banking, which affects their risk-taking relatively quickly, while shadow banking, after long-term operation and fermentation, accumulates riskiness and vulnerability and is prone to backfire and contagion in the banking system in the long run, supporting Hypothesis 2.

In an analysis of variance decomposition of central bank written communication (*cinei*), it is worth noting the contribution of its own (*cinei*) and the duration of accommodative monetary policy (*d_plmp*). ① The contribution of its own lagged one period decreases relatively quickly from 17.7% in the first period to 11.5% in the second period, to 10.3% in the fifth period and gradually stabilizes, while combined with the third graph in the third row of Fig 1. It shows that there is a strong correlation and succession between the adjacent written communication of the central bank before and after. The textual difference pairs are smaller, but the degree of influence on written communication decreases after two periods, which is consistent with the characteristics and patterns of the Monetary Policy Implementation Report issued by the People’s Bank of China. ② The contribution of the duration of accommodative monetary policy (*d_plmp*) to the central bank’s written communication index (*cinei*), although declining, has always occupied an important proportion, gradually declining from the initial 61.5% to 30.9% and stabilizing. This shows that the accommodative macroeconomic conditions and policy environment clearly influence the central bank’s communication and exchange, especially the short-term effect, which is extremely significant. The reality is clearly such that the Monetary Policy Implementation Report is indeed a summary of the monetary policy and macroeconomic conditions of the approaching period and a look at future policy and economic development prospects based on the recent domestic and foreign macro policy environment. The duration of the easing policy and economic conditions at the time of the Report’s writing plays an absolutely dominant position in influencing the content of the Report, which supports the first half of the central bank communication and exchange mechanism of the monetary policy bank risk-taking channel proposed by Blinder [30] and Borio and Zhu [5]; in other words, easing monetary policy influences central bank communication and exchange.

### 5.2. Robustness tests

To ensure the robustness of Model (1), various empirical tests such as substituting bank risk-taking variables, considering central bank communication on foreign policy and economic conditions and changing the order of the main variables, are carried out, and the PVAR2 method is used in these tests. Unlike the PVAR approach in the main regression, the first variable appearing in the impulse response plot under the PVAR2 approach is the response variable and the second variable is the shock variable. The robustness tests are all in general agreement with the main regression, and the results are not presented due to space constraints. Test results are available from the author on request. It is worth noting that the central bank foreign communication index (*ciwai*) negatively affects bank risk-taking (*rndd*), suggesting that bank risk-taking also responds to the Report’s communication and exchange on the macro environment in other countries, with a lower central bank foreign communication index (*ciwai*) representing a relatively poor foreign policy situation, in contrast to a potentially relatively accommodative domestic policy, which would clearly stimulate domestic commercial bank risk-taking, confirming Hypothesis 1 in reverse.

### 5.3. Test results and analysis based on the heterogeneity of banks

**Fig 2:**
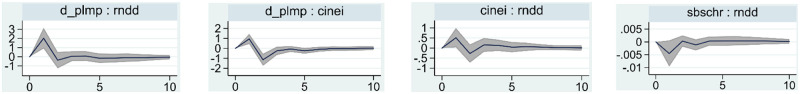
200 Monte-Carlo impulse response plots for Model (2) under small and medium-sized joint-stock banks.

**Fig 3:**
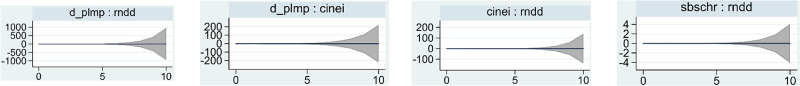
200 Monte-Carlo impulse response plots for Model (3) under large state-owned banks.

The previous section has explained that banks may differ in their risk-taking capacity depending on their size and nature of ownership. Therefore, we divide banks into large state-owned banks (Bank of China, Agricultural Bank of China, Industrial and Commercial Bank of China, China Construction Bank, Bank of Communications, and Postal Savings Bank of China). The PVAR model for the sample of small and medium-sized joint-stock banks is denoted as Model (2). The PVAR model for the sample of large state-owned banks is denoted as Model (3) when the bank risk-taking proxy variable is the proportion of nondebt deposits (*rndd*). Due to space limitation, its unit root test, lag selection test, smoothness test, Granger causality test and variance decomposition analysis are not listed, and only the impulse response plots of the key parts are given (see Figs 2 and 3).

It is easy to see that the impulse response plots of small and medium-sized joint-stock banks (Fig 2) are largely consistent with those of the full sample of banks (Fig 1), but the positive response of risk-taking to central bank communication is more pronounced for small and medium-sized joint-stock banks. According to the third panel in the first column of Fig 2, the coefficient of the positive effect of central bank communication on banks’ risk-taking in the first period is 0.5112 (according to the GMM results under the PVAR approach) greater than 0.4175 under the full sample in Model (1).

However, the impulse responses of the large bank sample (Fig 3) GMM results under the PVAR method are not significant. The system is not stable and diverges significantly from the full sample of banks for the following possible reasons. ① Large state-owned banks have more channels to obtain time-sensitive information. The six major banks of China, Agriculture, Industry, Construction and Communications and Post and Reserve are naturally related to the central bank and have diversified. More rapid communication channels with the government and monetary authorities and access to information are less dependent on the central bank for communication than small and medium-sized joint-stock banks. ② Large state-owned banks mainly implement policies mainly and have lower information sensitivity. Since the founding of the country, large state-owned banks have been separated from the People’s Bank of China by their mother’s womb, shouldering the responsibility and cost of reform and transformation, their business behavior has been highly consistent with national policies and the relatively lagging Monetary Policy Implementation Report has had little impact on them. ③ The demonstration effect requires large state-owned banks to be transparent and legal. Due to the industry’s model responsibility and social responsibility, large state-owned banks are highly transparent and regulated, and their operations, including shadow banking, are relatively standardized and legal. Therefore, the impact of large state-owned banks’ risk-taking on their own risk-taking is inevitably smaller than the impact of nonstandardized and hidden shadow banking developed by small and medium-sized joint-stock banks. ④ The scale effect weakens the impact of shadow banking. Large state-owned banks are market leaders with absolute market positions and bargaining power, which weaken the influence of shadow banking on their bank risk-taking.

The above supports Hypothesis 3 that the impact of central bank communication on bank risk-taking in the presence of shadow banking is more pronounced among small and medium-sized joint-stock banks than among large state-owned banks.

## 6. Further analysis

Based on the above analysis, it is necessary to further explore the effect of the bank risk-taking channel under the growth rate of central bank written communication and shadow banking. To test what effect central bank communication can have on economic performance through the bank risk-taking channel in the context of the development of shadow banking, and to this end, we construct the corresponding PVAR empirical model in the following form:

$$X_{it}=\beta_{i}+\sum_{j=1}^{p}B_{j}X_{i,t-j}+d_{t}+f_{i}+\varepsilon_{it}$$ (22)

i∈{1,2,……,N},t∈{1,2,……,T}

where X_it is a 1 × m-dimensional vector of six variables at year t of the i-th bank, expanded as

Xit=EPEtSBtCItBRTitRISKitEBit/SAit (23)

Compared with Eq (19), a variable *EB*_*it*_/*SA*_*it*_ is added here. *EB*_*it*_ represents the profitability of banks, which is generally measured by the return on total assets or net assets, but the return on assets in the case of high leverage is volatile and easily distorted, which is not conducive to the study. Compared with the return on total assets, the ratio of noninterest income can better reflect the result of banks’ active risk-taking, so the ratio of noninterest income (*d_nni*) is selected as a proxy variable for bank profitability. The noninterest income share (*nni*) refers to the proportion of operating income other than the bank’s carry income to operating income. Noninterest income includes income from activities such as intermediation, consulting, and investment, where *d_nni* is the first-order difference of the variable *nni*, and its P value for both LLC and IPS unit root tests is significantly zero at the 1% level. *SA*_*it*_ represents the asset size of banks, and the logarithm of the total asset size of banks (*tacb*) is used as a proxy variable. Specifically, it is divided into the four models in Table 7 below.

**Table 7:** Description of the PVAR2 model for further testing.

Model (4)	Model (5)	Model (6)	Model (7)
*d_plmp*	*d_plmp*	*d_plmp*	*d_plmp*
*sbschr*	*sbschr*	*sbschr*	*sbschr*
*cinei*	*cinei*	*cinei*	*cinei*
*car*	*car*	*car*	*car*
*rndd*	*nlr*	*rndd*	*nlr*
*d_nni*	*d_nni*	*tacb*	*tacb*

**Fig 4:**
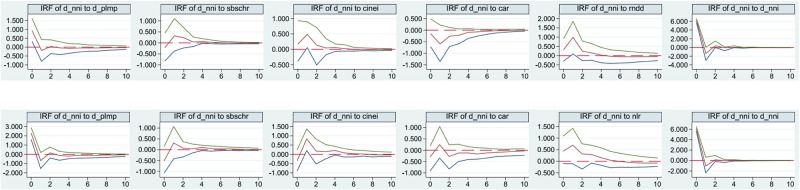
Impulse response of bank noninterest income (*d_nni*) to each factor.

Due to the space limitations, the Models’ (4)-(7) data smoothness test, determination of the optimal lag order, stability test, Granger causality test, and variance decomposition results in the further analysis are omitted, and only the impulse response analysis plots of the key parts of the four models are given. Figs 4 and 6 show the impulse responses of bank earnings and bank size to each factor, respectively, focusing on how shocks to bank risk-taking (*rndd* and *nlr*) cause changes in the response of the share of bank noninterest income (*d_nni*) and bank asset size (*tacb*).

**Fig 5:**
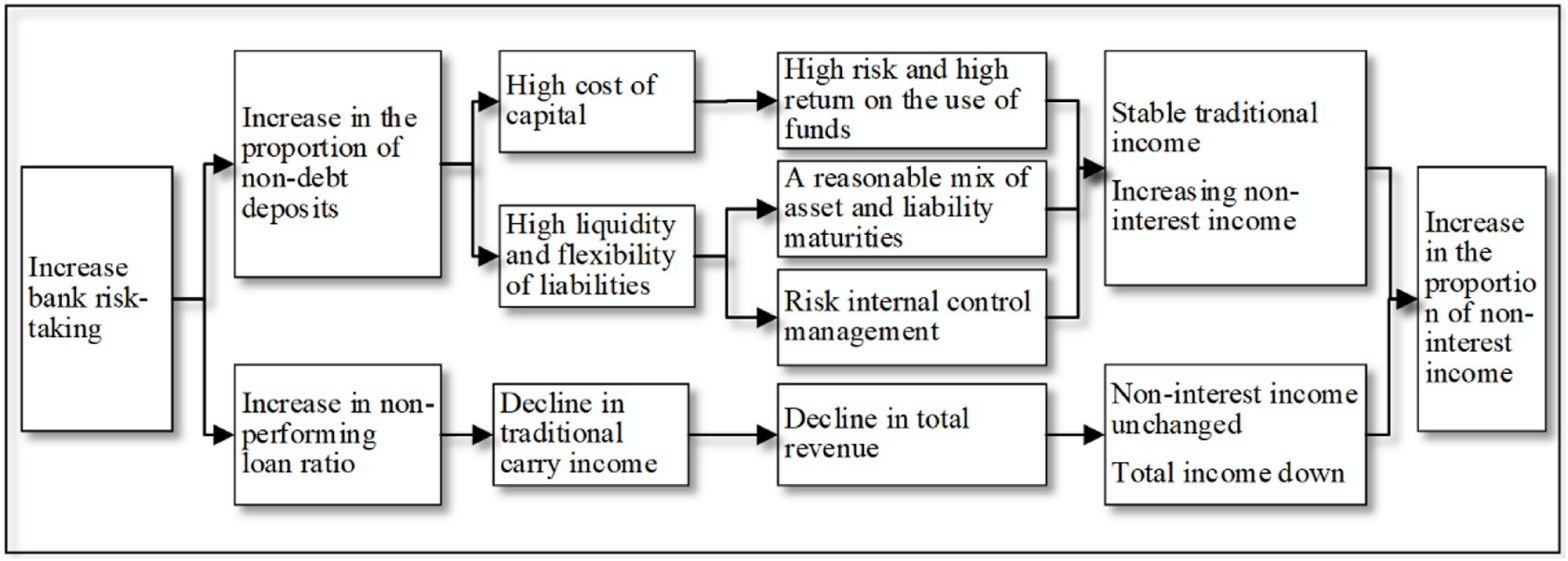
Mechanisms by which bank risk-taking affects the share of non-interest income of banks.

In Fig 4, both the ratio of nondeposit liabilities (*rndd*) and the nonperforming loan ratio (*nlr*) of banks hit the ratio of noninterest income (*nni*) of banks in the same direction in the same period, reaching a maximum in the first period and then gradually decreasing, returning to zero and remaining stable in the fourth and fifth periods. This shows that the increase of banks’ risk-taking causes banks’ profitability to improve, mainly due to the following reasons (see Fig 5). The higher the proportion of nondeposit liabilities of banks, the higher their funding costs tend to be, and the use of funds is bound to be more in pursuit of high returns. The high flexibility and liquidity of nondeposit liability funding sources make it easier to match the maturity and comprehensive management of assets and liabilities, which in turn makes the allocation of banks’ asset portfolios more reasonable, and increases the sources of noninterest income under the relatively stable situation of banks’ traditional carry income, thus causing the proportion of banks’ noninterest income to increase. An increase in the NPL ratio affects the bank’s interest income, which, other things being equal, decreases, resulting in a higher share of noninterest income.

**Fig 6:**
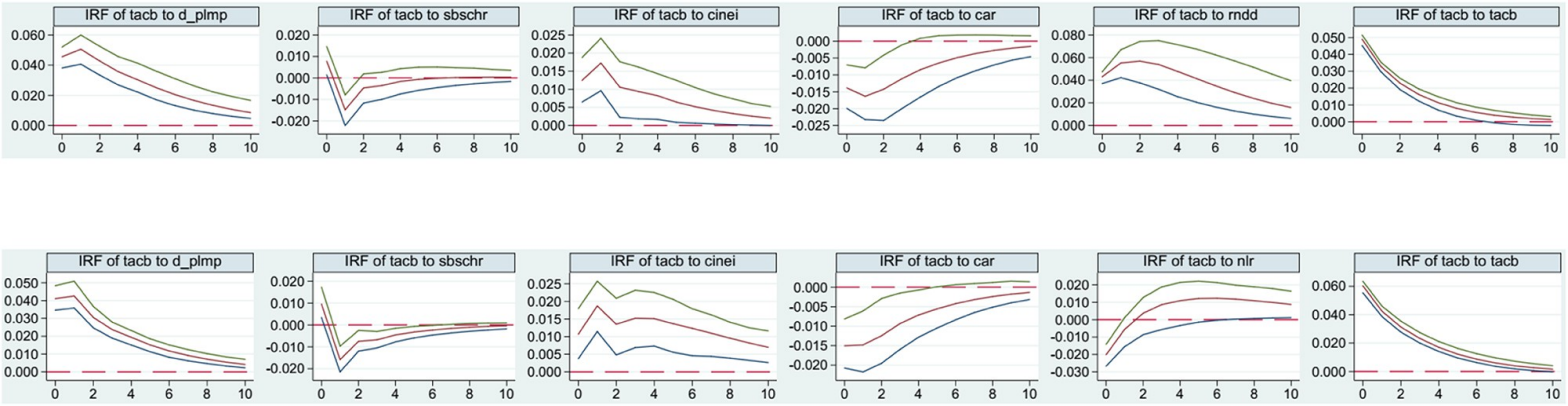
Impulse response of bank asset size (tacb) to each factor.

In Fig 6, the share of nondebt deposits (*rndd*) of banks positively shocks the total asset size of banks (*tacb*) in the immediate period and gradually decreases after reaching a maximum around the third period, but still maintains a positive relationship and remains stable. The bank nonperforming loan ratio (*nlr*) shocks the total bank asset size (*tacb*) negatively in the immediate period, but rises to a positive relationship around the second period and holds steady. The difference between the two models is related to banks’ active and passive risk-taking. Generally, a higher percentage of nondeposit liabilities of banks tends to mean that banks are more advanced in their operating concepts and methods and have a stronger ability to actively obtain funds (active liabilities), although the risk-taking is greater compared to deposit funds (passive liabilities), but the relatively stable deposit size does not easily restrict business development. It is conducive to the diversification of assets and expansion of scale, thus leading to the expansion of immediate bank size. The bank’s passive risk taking represented by the non-performing loan rate is not quite the same. When the nonperforming loan rate rises, it may be due to certain problems in the bank’s management and risk control, or it may be due to the deterioration of the macroeconomic environment and the conditions of microenterprises and individuals, all of which will cause the bank to fail to recover the principal and interest on schedule. At the same time, it requires more special loss provisions, resulting in a reduction in the funds available to banks in the immediate period. Thus, in a short period of time, the bank’s passive risk taking (*nlr*) will cause a decline in the size of bank assets. However, when banks realize such problems, they will certainly update and improve their management and risk management levels, or macro policies will also be regulated to release more lower-cost liquidity, stimulating banks to use funds more prudently. Thus, banks’ passive risk-taking will still lead to asset size expansion in the long run. Therefore, the overall increase in bank risk-taking (*rndd* and *nlr*) leads to the expansion of bank size, which supports Ayele’s [73] argument that bank risk-taking behavior is the Granger cause of bank size.

## 7. Conclusions and recommendations

Central bank communication is widely used in the macroeconomic regulation of monetary policy. As an extremely important and frequently operated unconventional monetary policy tool, how central bank communication should reasonably serve the monetary policy transmission mechanism, act on the risk-taking of financial entities represented by commercial banks and enhance the effectiveness of monetary policy is an important challenge in building a leading modern central banking system and preventing and resolving systemic financial risks in China. At the same time, with the development of domestic financial innovation, shadow banking has flourished, effectively solving the problem of complex financing for some SMEs, but its scale expansion can also affect the operation of banks, especially in risk-taking. Therefore, this paper studies the relationship between central bank communication, shadow banking, and bank risk-taking based on the central bank’s most formal and authoritative Monetary Policy Implementation Report and further analyzes the possible economic consequences. The theoretical model and empirical tests find that ① the central bank’s written communication index positively affects bank risk-taking, verifying the existence of a central bank communication and exchange mechanism for the monetary policy bank risk-taking channel. ② The development of shadow banking may lead to a decline in banks’ risk-taking in the short term, but there is a stable positive correlation between the two in the long term. ③ The above findings are supported in robustness tests, and central bank communication on foreign policy and the economic environment is negatively related to bank risk-taking. ④ The above results are heterogeneous across ownership systems. In other words, they are more pronounced in small and medium-sized joint-stock banks and not significant in large state-owned banks. ⑤ Further research proves that bank risk-taking positively affects banks’ return profiles and asset size.

In response to the above findings, the following policy recommendations are made. ① A good written communication strategy should constantly seek a more moderate frequency, neutral expression, and objective tone. In this way, banks and other market players can clearly understand the policy intentions of monetary authorities without creating overly optimistic expectations. Of course, this is difficult, and the monetary authorities also need to use other communication methods based on the nature of ownership (state-owned and joint-stock) and the size (large and small and medium-sized) of banks. For example, new social means such as central bank working papers, press conferences, speeches by central bank governors and officials, and official WeChat public numbers can soften the rigid expression of the Report and mitigate the lagging effect. This could provide the market with a clearer basis for decision-making. ② At the same time, the central bank should also pay attention to enhancing communication in the intricate global environment and major national policies to facilitate banks’ comprehensive understanding of the domestic and international situation and the attitude and position of the PBOC for scientific decision-making. ③ For shadow banking, while encouraging formal operations and legal and orderly development, it should also strengthen supervision and establish a targeted monitoring system, such as monitoring and guiding its fund flow areas. It should also try to let it serve small and microenterprises and high-tech enterprises that are difficult for formal finance to function in the short term. A perfect supervision system and transparent use of funds can effectively reduce the driving effect of shadow banking on banks’ risk-taking channels and facilitate risk prevention and resolution in the banking system. ④ In addition, the increase in banks’ risk-taking is not necessarily bad and may help banks increase the proportion of noninterest income and asset size. Banks should constantly strengthen their thinking on forward-looking central bank communication, global planning, and strategic layout, including shadow banking within a manageable degree of risk, to develop safety, quality, efficiency, and scale speed.
